# Diabetes and chronic kidney disease in Chinese adults: a population-based cohort study

**DOI:** 10.1136/bmjdrc-2023-003721

**Published:** 2024-01-24

**Authors:** Xue Wang, Lu Chen, Kexiang Shi, Jun Lv, Dianjianyi Sun, Pei Pei, Ling Yang, Yiping Chen, Huaidong Du, Jiaqiu Liu, Xiaoming Yang, Maxim Barnard, Junshi Chen, Zhengming Chen, Liming Li, Canqing Yu

**Affiliations:** 1 Department of Epidemiology and Biostatistics, School of Public Health, Peking University, Beijing, China; 2 Peking University Center for Public Health and Epidemic Preparedness & Response, Beijing, China; 3 Key Laboratory of Epidemiology of Major Diseases (Peking University), Ministry of Education, Beijing, China; 4 Medical Research Council Population Health Research Unit at the University of Oxford, Oxford, UK; 5 Clinical Trial Service Unit & Epidemiological Studies Unit (CTSU), Nuffield Department of Population Health, University of Oxford, Oxford, UK; 6 NCDs Prevention and Control Department, Pengzhou CDC, Pengzhou, China; 7 China National Center for Food Safety Risk Assessment, Beijing, China

**Keywords:** Chronic Kidney Disease, Diabetes Mellitus, Cohort Studies

## Abstract

**Introduction:**

Cohort evidence of the association of diabetes mellitus (DM) with chronic kidney disease (CKD) is limited. Previous studies often describe patients with kidney disease and diabetes as diabetic kidney disease (DKD) or CKD, ignoring other subtypes. The present study aimed to assess the prospective association of diabetes status (no diabetes, pre-diabetes, screened diabetes, previously diagnosed controlled/uncontrolled diabetes with/without antidiabetic treatment) and random plasma glucose (RPG) with CKD risk (including CKD subtypes) among Chinese adults.

**Research design and methods:**

The present study included 472 545 participants from the China Kadoorie Biobank, using baseline information on diabetes and RPG. The incident CKD and its subtypes were collected through linkage with the national health insurance system during follow-up. Cox regression models were used to calculate the HR and 95% CI.

**Results:**

During 11.8 years of mean follow-up, 5417 adults developed CKD. Screened plus previously diagnosed diabetes was positively associated with CKD (HR=4.52, 95% CI 4.23 to 4.83), DKD (HR=33.85, 95% CI 29.56 to 38.76), and glomerulonephritis (HR=1.66, 95% CI 1.40 to 1.97). In those with previously diagnosed diabetes, participants with uncontrolled diabetes represented higher risks of CKD, DKD, and glomerulonephritis compared with those with controlled RPG. The risk of DKD was found to rise in participants with pre-diabetes and increased with the elevated RPG level, even in those without diabetes.

**Conclusions:**

Among Chinese adults, diabetes was positively associated with CKD, DKD, and glomerulonephritis. Screen-detected and uncontrolled DM had a high risk of CKD, and pre-diabetes was associated with a greater risk of DKD, highlighting the significance of lifelong glycemic management.

**WHAT IS ALREADY KNOWN ON THIS TOPIC**

- Diabetes mellitus is the primary driver of chronic kidney disease (CKD), with the prevalence of CKD and diabetes (including pre-diabetes) increasing in China.
- Previous studies underexplored medical perspectives on the differences of diabetes with CKD subtypes, and the association between pre-diabetes and CKD remains uncertain.

**WHAT THIS STUDY ADDS**

- Adopting a longitudinal study design, we found that diabetes was independently associated with an increased risk of CKD and specific subtypes, including diabetic kidney disease (DKD) and non-diabetic kidney disease (NDKD) subtype—glomerulonephritis.
- Glycemic control, either through antidiabetic treatment or other self-management approaches, could partly offset the risk of CKD brought by diabetes in patients with diagnosed diabetes.
- The risk of DKD was found to rise in participants with pre-diabetes and increased with the elevated random plasma glucose level, even in those without diabetes.

**HOW THIS STUDY MIGHT AFFECT RESEARCH, PRACTICE OR POLICY**

- Our data highlighted the management of patients with diabetes for DKD and NDKD prevention.
- Early screening of CKD, broadening high-risk populations from diabetes to pre-diabetes, was worth investigating.

## Introduction

Chronic kidney disease (CKD) accounts for a heavy global health burden due to its high prevalence and subsequent risk of end-stage renal disease, cardiovascular disease, and premature death.^1^ The global burden of CKD is rapidly increasing, and it is estimated to be the fifth leading cause of years of life lost worldwide by 2040.^2^ There is a growing awareness of the importance of identifying high-risk individuals to prevent CKD and its complications. Given that diabetes mellitus (DM) is the primary driver of CKD, preventing CKD in people with diabetes could have a significant impact on lowering the global CKD burden.^3^

There are five major subtypes of CKD, including diabetic nephropathy, hypertensive renal disease, glomerular disease, renal tubulointerstitial diseases, and obstructive nephropathy.^4^ However, previous studies often define patients with DM with CKD as diabetic nephropathy or simply CKD, leading to underexplored medical perspectives on diabetes with other CKD subtypes.^5^

Furthermore, it has been reported that up to one-third of patients with newly diagnosed diabetes already had kidney damage, implying that the effect of hyperglycemia on the occurrence of CKD may initiate before glucose levels reach the diagnostic threshold.^6 7^ Nevertheless, the effect of non-diabetic hyperglycemia, namely pre-diabetes, remains largely unclear. Clarifying its link with CKD is critical for clinical practice and public health. Broadening the high-risk population from diabetes to pre-diabetes in the guideline for early screening of CKD may represent an early window of opportunity for prevention.

In recent decades, China has seen a marked increase in diabetes and pre-diabetes prevalence.^8^ However, there is little reliable prospective evidence of their effect on the risk of CKD in China. Therefore, we investigated the associations of diabetes and blood glucose levels with CKD risk, including its subtypes, in the China Kadoorie Biobank (CKB) population of 0.5 million adults.

## Research design and methods

### Study population

The CKB study is an ongoing population-based prospective cohort study, and the study’s design and methods have been reported previously.^9 10^ 512 724 adults aged 30–79 years were recruited at baseline from June 2004 to July 2008. Participants with previously diagnosed kidney disease (n=7574) and major diseases that may alter glucose metabolism and intercorrelate with CKD, namely cancer (n=2527), coronary heart disease (n=14 737), and stroke (n=7453), were excluded. Those with missing body mass index (BMI) (n=2) or plasma glucose (n=7885) and those who lost their follow-up immediately after the baseline survey (n=1) were further excluded, leaving 472 545 participants for the primary analyses. All participants provided their written informed consent.

### Assessment of diabetes

At the baseline survey, all participants were asked ‘Has a doctor ever told you that you had had diabetes?’. Individuals reporting a history of diabetes were further asked to provide additional information about age at diagnosis and current diabetes medications.

A non-fasting blood sample of 10 mL was collected from the participant into an EDTA vacutainer (BD Hemogard, BD, USA), with the time since the participant last ate being recorded. The random plasma glucose (RPG) levels were tested on-site using the SureStep Plus System (Johnson & Johnson). The tests were regularly calibrated with the manufacturer’s quality solution. Participants with RPG levels ≥7.8 and <11.1 mmol/L were invited to return for a fasting plasma glucose (FPG) test the next day.^9^ For consistency, we used only the first single RPG measurement in the RPG-related analysis.

In the present study, participants who reported diabetes history were defined as having previously diagnosed diabetes. Those without a history of diabetes were considered to have screen-detected diabetes if they met any of the following criteria: (1) RPG≥7.0 mmol/L if the time since the last eating was ≥8 hours; (2) ≥11.1 mmol/L if the time since the last eating was <8 hours; (3) an FPG≥7.0 mmol/L on subsequent testing.^11 12^ According to the medication and blood glucose level, we further categorized the participants with previously diagnosed diabetes into four groups: controlled diabetes without treatment, uncontrolled diabetes without treatment, controlled diabetes under treatment, and uncontrolled diabetes under treatment (online supplemental figure 1).

10.1136/bmjdrc-2023-003721.supp1 Supplementary data

### Identification of CKD cases

The outcomes were ascertained through linkage with local disease registries, death registries, and the national health insurance system and were supplemented by active follow-up. Each event was coded by health workers blinded to baseline characteristics of participants according to the International Classification of Diseases, Tenth Revision (ICD-10). In the present study, participants were followed up from the time they completed the baseline survey until the occurrence of CKD, death, loss to follow-up, or December 31, 2018, whichever came first. The ICD-10 codes for CKD cases were adapted from the China Kidney Disease Network,^4^ which included the five main subtypes. Detailed ICD-10 codes were listed in online supplemental table 1.

### Assessment of covariates

At the baseline, participants had a face-to-face interview using a laptop-based questionnaire collecting information on sociodemographic characteristics (eg, age, sex, education, household income, and occupation), smoking status, alcohol consumption, physical activity, and medical history (hypertension, diabetes, kidney disease, cancer, coronary heart disease, and stroke).

Trained staff measured standing height (cm), body weight (kg), and blood pressure (mm Hg) using calibrated instruments with standard procedures. Participants were measured in light clothes for body weight and waist circumference and without shoes for standing height. The daily level of physical activity was calculated by multiplying the metabolic equivalents of task (METs) value for a particular type of physical activity by the hours spent on that activity per day and summing the MET-hours for all activities (MET-hours/day). BMI was calculated as weight (kg)/height (m).^2^ Prevalent hypertension was defined as systolic blood pressure ≥140 mm Hg, diastolic blood pressure ≥90 mm Hg, self-reported diagnosis of hypertension, or self-reported use of antihypertensive drugs at baseline.

### Statistical analysis

Baseline characteristics of participants were presented as age, sex, and region-adjusted means or percentages in each group of diabetes status, using linear regression models for continuous variables and logistic models for categorical variables, respectively.

Cox proportional hazards models were used to estimate adjusted HRs of CKD associated with diabetes status at baseline, stratified by age at risk (5-year groups) and study area (10 areas), and adjusted for sex (male, female), educational level (no formal school, primary school, middle school, high school, technical school or college, university), household income (<¥20 000, ≥¥20 000/year), and occupation (manual, others) in model 1; additionally adjusted for alcohol consumption (never or occasional drinker, former drinker, weekly drinker, daily drinker: <15, 15–30, 30–60, ≥60 g/day) and cigarette consumption (never or occasional smoker, former smoker, current regular smoker: <15, 15–25, ≥25 cigarettes or equivalents/day), physical activity (MET-hours/day, continuous) in model 2; additionally adjusted BMI (kg/m^2^, continuous) and hypertension status (yes or no) in model 3. HRs associated with CKD subtypes were estimated using consistent models, with competing risks of other subtypes not considered here.

With additional adjustments for the fasting time in models, we analyzed the association between RPG and CKD and its subtypes. The RPG levels were categorized into four groups (<5.6 (reference), 5.6–6.9, 7.0–11.0, and ≥11.1 mmol/L), considering the FPG thresholds for impaired fasting glucose and diabetes.^11 12^ RPG was also treated as a continuous variable, and HR (95% CI) for 1 mmol/L increment of RPG was estimated. Furthermore, we used restricted cubic splines with four knots at the 5th, 35th, 65th, and 95th percentiles of RPG to flexibly model the association of RPG with CKD.

Sensitivity analyses were performed to test the robustness of the results. First, we additionally adjusted for diabetes treatment. Second, CKD cases within the initial 2 years of follow-up were excluded to minimize the possibility of reverse causality. Third, additional analysis of the associations of diabetes with CKD subtypes was censored at the earliest dates for any incidence of CKD subtypes to exclude the effect of the incidence of subsequent subtypes. Lastly, we analyzed the association between RPG and CKD among participants who had not been previously diagnosed with diabetes and those fasting for ≥8 hours at baseline.

All statistical analyses were performed using Stata V.15.0, and statistical significance was set at a two-tailed p<0.05.

## Results

Among 472 545 participants, the average age was 51.6±10.6 years at baseline, 41.0% were male, and 56.3% were from rural areas. At baseline, 12 745 (2.7%) participants reported a history of diabetes (previously diagnosed diabetes), and 12 792 (2.7%) were screen detected by the blood test. Participants with diabetes were more likely to be old, female, urban residents, with higher RPG, waist circumference, and a history of hypertension, and were less likely to be manual workers. Compared with the diagnosed diabetes, patients with screened diabetes were more likely to be rural residents, farmers or workers, current weekly drinkers, and with higher RPG levels (table 1).

**Table 1:** Baseline characteristics by diabetes status in CKB (n=472 545)

	No diabetes*	Diabetes*		
		Total	Screen detected	Previously diagnosed
Participants (n)	447 008	25 537	12 792	12 745
Age (years)	51.2	57.3	56.0	58.5
Female (%)	58.8	62.8	61.7	63.8
Urban resident (%)	43.0	57.5	53.9	60.8
>6 years of education (%)	49.4	49.3	47.3	51.2
Household income ≥¥20 000/year (%)	42.8	44.5	43.7	45.2
Worker or farmer	57.6	48.8	52.5	44.7
Current regular smoker† (%)				
Male	67.9	66.3	68.2	64.4
Female	2.6	2.6	2.5	2.7
Current weekly drinker (%)				
Male	33.9	30.0	36.8	22.9
Female	2.1	1.0	1.6	0.6
Physical activity (MET-hours/day)	21.6	19.6	20.3	18.8
BMI (kg/m^2^)	23.5	24.8	25.0	24.5
Waist circumference (cm)	79.8	84.6	85.0	84.1
RPG (mmol/L)	5.7	12.2	13.0	11.3
Hypertension (%)	32.9	49.3	49.3	48.9

Results were adjusted for age, region, and sex (where appropriate).

*The comparisons between the no diabetes and diabetes groups were significant at p<0.01 except for education and female smoking.

†Former smokers who quitted smoking due to illness were counted as current smokers.

BMI, body mass index; CKB, China Kadoorie Biobank; MET, metabolic equivalent of task; RPG, random plasma glucose.

### Associations of diabetes with risk of CKD

During a median follow-up of 11.8 years, a total of 5417 CKD cases were documented, including 1172 cases of diabetic nephropathy, 1531 cases of glomerular disease, 364 cases of hypertensive renal disease, 480 cases of renal tubulointerstitial diseases, 595 cases of obstructive nephropathy, and 525 cases of other subtypes. After adjusting for multiple variables, diabetes was found to be positively associated with an increased risk of CKD (HR=4.52, 95% CI 4.23 to 4.83), with the strongest association observed in diabetic nephropathy (HR=33.85, 95% CI 29.56 to 38.76), and a significantly elevated risk in glomerulonephritis (HR=1.66, 95% CI 1.40 to 1.97), but not for other types of CKD (table 2). The associations of diabetes with CKD, diabetic nephropathy, and glomerulonephritis attenuated with additional adjustment for diabetes treatment but persisted in all the sensitivity analyses (online supplemental table 2).

**Table 2:** Adjusted HRs for CKD by diabetes status at baseline

	Events (n)	Incidence rate*	HR (95% CI)		
			Model 1	Model 2	Model 3
CKD					
No diabetes	4116	78.28	1.00	1.00	1.00
Diabetes	1301	468.75	5.19 (4.86–5.54)	5.09 (4.77–5.44)	4.52 (4.23–4.83)
Diabetic nephropathy					
No diabetes	348	6.60	1.00	1.00	1.00
Diabetes	824	295.33	38.71 (33.89–44.21)	37.82 (33.10–43.21)	33.85 (29.56–38.76)
Glomerulonephritis					
No diabetes	1372	26.05	1.00	1.00	1.00
Diabetes	159	56.67	1.89 (1.59–2.23)	1.86 (1.57–2.20)	1.66 (1.40–1.97)
Hypertensive nephropathy					
No diabetes	320	6.07	1.00	1.00	1.00
Diabetes	44	15.66	1.79 (1.29–2.47)	1.75 (1.27–2.43)	1.37 (0.99–1.90)
Tubulointerstitial nephritis					
No diabetes	437	8.29	1.00	1.00	1.00
Diabetes	43	15.31	1.44 (1.04–1.98)	1.42 (1.03–1.96)	1.34 (0.97–1.85)
Obstructive nephropathy					
No diabetes	573	10.87	1.00	1.00	1.00
Diabetes	22	7.83	0.94 (0.61–1.45)	0.92 (0.60–1.42)	0.86 (0.56–1.32)

HRs were adjusted for sex, education, household income, and occupation in model 1 and stratified by study regions and age at the study date; model 2 adjusted for variables in model 1 plus alcohol consumption, smoking consumption, and MET; model 3 adjusted for variables in model 2 plus BMI and hypertension status.

*The incidence rate was derived from CKD case count divided by total person-years (presented as per 100 000 person-years). Total person-year at risk was calculated from the time participants completed the baseline survey until the occurrence of CKD, death, loss to follow-up, or December 31, 2018, whichever came first.

BMI, body mass index; CKD, chronic kidney disease; MET, metabolic equivalent of task.

Besides, an elevated risk of CKD was observed in individuals with screened-detected diabetes (HR=2.83, 95% CI 2.55 to 3.15). In terms of diabetes management, the adjusted HRs of CKD were lowest in the controlled diabetes without medication group (HR=2.21, 95% CI 1.50 to 3.25), while were highest in the uncontrolled diabetes receiving antidiabetic treatment group (HR=8.17, 95% CI 7.46 to 8.95). Patients with DM with controlled blood glucose levels exhibited lower risks of CKD than those with hyperglycemia status whether were treated or not (online supplemental table 3).

### Associations of RPG with risk of CKD

**Figure 1:**
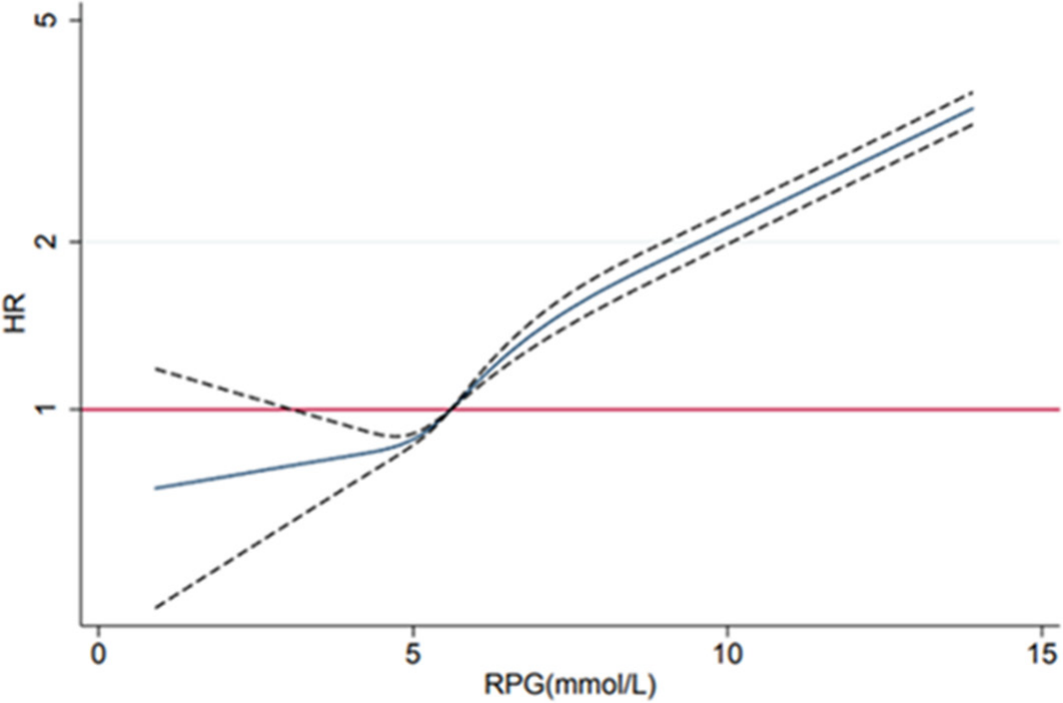
Association of random plasma glucose (RPG) with chronic kidney disease (CKD) risk. RPG was included as restricted cubic spline terms in the Cox model, using four knots at the 5th, 35th, 65th, and 95th percentiles. HRs were adjusted for sex, education, household income, occupation, fasting time, alcohol consumption, smoking consumption, physical activity, body mass index (BMI), and hypertension status and stratified by study regions and age at the study date. The reference RPG was 5.6 mmol/L.

Compared with the RPG level of <5.6 mmol/L, those with RPG levels of 5.6–6.9, 7.0–11.0, and ≥11.1 mmol/L exhibited an augmented risk of CKD, with adjusted HRs of 1.19 (1.11–1.27), 1.79 (1.66–1.94), and 6.63 (6.09–7.22), respectively. The association was in a dose–response manner with HR=1.15 (95% CI 1.14 to 1.15) for every 1 mmol/L increase in RPG. Similar trends were also observed in diabetic nephropathy and glomerulonephritis (table 3). Multivariable-adjusted restricted cubic spline analysis indicated a ‘J-shaped’ association of RPG with CKD (p value for non-linearity <0.001) (figure 1). In the sensitivity analyses, the results remained essentially unchanged after excluding participants with previously diagnosed diabetes (online supplemental table 4). The diabetic kidney disease (DKD) risk persisted but CKD risk became insignificant for those with RPG of 5.6–6.9 mmol/L when limited to those fasting for ≥8 hours (online supplemental table 5).

**Table 3:** Adjusted HRs for CKD by levels of RPG

	Events (n)	Incidence rate*	HR (95% CI)		
			Model 1	Model 2	Model 3
CKD (mmol/L)					
<5.6	2066	68.58	1.00	1.00	1.00
5.6–6.9	1480	88.14	1.22 (1.14–1.31)	1.22 (1.14–1.31)	1.19 (1.11–1.27)
7.0–11.0	1014	144.29	1.94 (1.79–2.10)	1.93 (1.79–2.09)	1.79 (1.66–1.94)
≥11.1	857	607.05	7.77 (7.15–8.45)	7.63 (7.01–8.30)	6.63 (6.09–7.22)
P value for trend			<0.001	<0.001	<0.001
Effect per 1 mmol/L			1.15 (1.15–1.16)	1.15 (1.15–1.16)	1.15 (1.14–1.15)
Diabetic nephropathy					
<5.6	153	5.07	1.00	1.00	1.00
5.6–6.9	165	9.80	1.93 (1.54–2.40)	1.93 (1.54–2.41)	1.86 (1.49–2.32)
7.0–11.0	306	43.40	8.45 (6.91–10.32)	8.39 (6.86–10.24)	7.67 (6.27–9.37)
≥11.1	548	385.85	68.69 (56.89–82.94)	66.88 (55.38–80.77)	57.98 (47.92–70.15)
P value for trend			<0.001	<0.001	<0.001
Effects per 1 mmol/L			1.25 (1.24–1.26)	1.25 (1.24–1.26)	1.24 (1.24–1.25)
Glomerulonephritis					
<5.6	714	23.67	1.00	1.00	1.00
5.6–6.9	451	26.80	1.15 (1.02–1.29)	1.15 (1.02–1.29)	1.12 (0.99–1.26)
7.0–11.0	259	36.73	1.54 (1.33–1.79)	1.54 (1.33–1.79)	1.44 (1.24–1.67)
≥11.1	107	74.83	3.04 (2.46–3.75)	3.01 (2.44–3.71)	2.64 (2.14–3.27)
P value for trend			<0.001	<0.001	<0.001
Effects per 1 mmol/L			1.09 (1.08–1.11)	1.09 (1.08–1.11)	1.08 (1.07–1.10)
Hypertensive nephropathy					
<5.6	149	4.93	1.00	1.00	1.00
5.6–6.9	111	6.59	1.17 (0.91–1.50)	1.16 (0.91–1.49)	1.09 (0.85–1.40)
7.0–11.0	81	11.48	1.78 (1.35–2.37)	1.78 (1.34–2.36)	1.50 (1.13–1.98)
≥11.1	23	16.06	2.12 (1.35–3.33)	2.09 (1.33–3.28)	1.55 (0.99–2.44)
P value for trend			<0.001	<0.001	0.015
Effects per 1 mmol/L			1.07 (1.04–1.10)	1.07 (1.04–1.10)	1.05 (1.02–1.08)
Tubulointerstitial nephritis					
<5.6	240	7.95	1.00	1.00	1.00
5.6–6.9	154	9.15	0.98 (0.80–1.20)	0.98 (0.80–1.21)	0.96 (0.78–1.18)
7.0–11.0	62	8.79	0.93 (0.70–1.24)	0.93 (0.69–1.24)	0.88 (0.66–1.18)
≥11.1	24	16.77	1.67 (1.08–2.56)	1.65 (1.07–2.54)	1.53 (0.99–2.35)
P value for trend			0.054	0.059	0.139
Effects per 1 mmol/L			1.04 (1.00–1.07)	1.04 (1.00–1.07)	1.03 (1.00–1.07)
Obstructive nephropathy					
<5.6	295	9.77	1.00	1.00	1.00
5.6–6.9	204	12.12	1.14 (0.95–1.36)	1.14 (0.95–1.36)	1.12 (0.93–1.34)
7.0–11.0	86	12.19	1.18 (0.92–1.51)	1.18 (0.92–1.51)	1.13 (0.88–1.45)
≥11.1	10	6.96	0.75 (0.40–1.42)	0.74 (0.39–1.39)	0.68 (0.36–1.28)
P value for trend			0.903	0.866	0.554
Effects per 1 mmol/L			1.01 (0.97–1.05)	1.01 (0.97–1.05)	1.00 (0.96–1.04)

HRs were adjusted for sex, education, household income, occupation, and fasting time in model 1 and stratified by study regions and age at the study date; model 2 adjusted for variables in model 1 plus alcohol consumption, smoking consumption, and MET; model 3 adjusted for variables in model 2 plus BMI and hypertension status.

*The incidence rate was derived from CKD case count divided by total person-years (presented as per 100 000 person-years). Total person-year at risk was calculated from the time participants completed the baseline survey until the occurrence of CKD, death, loss to follow-up, or December 31, 2018, whichever came first.

BMI, body mass index; CKD, chronic kidney disease; MET, metabolic equivalent of task; RPG, random plasma glucose.

## Discussion

Based on a large nationwide prospective cohort, diabetes was independently associated with an increased risk of CKD, diabetic nephropathy, and glomerulonephritis. Such associations were also observed for screen-detected diabetes, as well as previously diagnosed diabetes, and participants with uncontrolled diabetes represented higher risk than those with controlled diabetes regardless of antidiabetic treatment status. Besides, the risk of DKD was found to rise in participants with pre-diabetes and RPG was also positively associated with CKD risk even in those without diabetes.

Two previous cohort studies have reported an increased risk of CKD in patients with diabetes, but neither of them differentiated between subtypes of CKD.^13 14^ It is important to distinguish CKD subtypes, especially between DKD and non-diabetic kidney disease (NDKD) for disease prevention and clinical practice.^15 16^ A 3-year prospective, multicenter cohort study found that patients with DKD had a higher risk of a worse prognosis than NDKD (HR=2.30, 95% CI 1.48 to 3.58).^17^ The present study is the first to explore the associations between DM and CKD subtypes and found that DM was associated with both DKD and NDKD (ie, glomerulonephritis) in Chinese adults. Studies show hyperglycemia is the core upstream driver of both DKD and NDKD, while the role of hyperglycemia is slightly different.^18^ In DKD, hyperglycemia directly leads to kidney lesions from many aspects, such as metabolism, inflammation, and hemodynamics, characterized by glomerular hyperfiltration, glomerular hypertension, hyper-reabsorption in the proximal tubule, glomerular hypertrophy, macroproteinuria, glomerulosclerosis, and then nephron loss.^18^ In NDKD, the loss of nephrons is caused by genetic or other acquired causes, and hyperglycemia further aggravates the damage to the kidney, which is an indirect cause of NDKD.^18^ This may partly explain the larger strength of association of DM with DKD than NDKD. 5.4% of the patients with CKD in CKB developed two or more subtypes of CKD. To avoid interactions between subtypes, we only retained the prior-onset subtype, and the results were not substantially changed.

In the present study, half of the diabetes were screen detected, close to rates reported in China (53.1%)^8^ and globally (47.9%).^19^ Screen-detected DM raised a significant public health issue due to its asymptomatic features and health risks.^20^ So far, only one Japanese retrospective study has evaluated the impact of screen-detected DM on the risk of developing DKD (HR=1.57, 95% CI 1.16 to 2.12).^21^ The present study revealed that screen-detected diabetes could increase the risk of CKD, DKD, and glomerulonephritis. Screened diabetes represented lower risks of CKD than uncontrolled diabetes previously diagnosed. Screened diabetes may be at an early stage of DM, where clinical symptoms have not manifested yet. Therefore, our findings implicated the importance of early diabetes screening for the prevention and management of CKD.

A Chinese nationwide population-based study reported that among individuals with diabetes, 67.8% remained untreated, and 50.8% of those treated had poor glycemic control.^22^ Similar to this study, our study revealed the prevalence of untreated diabetes was 57.7%, and 60.7% showed abnormal blood glucose after treatment. Higher risks of CKD were borne in uncontrolled diabetes and with additional adjustment for diabetes treatment, the risk of DKD and glomerulonephritis in patients with diabetes attenuated in the current study. The results highlighted the need for rigorous management of diabetes and optimal blood glucose control to mitigate the clinical and economic burden of CKD. We found the risk of CKD was slightly higher in patients with treated diabetes than in untreated ones. Antidiabetic medication adherence is important for maximizing the effectiveness of pharmaceutical therapy and the role of self-management, like maintaining healthy lifestyles, including diet habits and exercise, could not be neglected to improve the prognosis of diabetes.^23 24^

The association of pre-diabetes with the risk of kidney disease has not been consistent across studies. Several cross-sectional studies have observed an increased risk of CKD in the pre-diabetes population.^25 26^ A meta-analysis of nine cohort studies also reported that impaired fasting glucose was modestly associated with increased CKD risk.^6^ However, a study based on the Systolic Blood Pressure Intervention Trial found that impaired fasting glucose was not associated with CKD.^27^ Besides, Korean and Japanese prospective cohort studies reported elevated HbA1c, but not impaired fasting glucose, which was an independent predictor of incident CKD, indicating that using different blood glucose indicators may yield different results.^28 29^ In a Mendelian randomization study conducted in a Danish cohort, RPG was causally associated with diabetic nephropathy, which supported the idea that an elevated glucose level has a causal role in the pathogenesis of the microvascular disease. Consistent with the Danish study, we observed a stepwise increase in the risk of CKD with increasing RPG within the normoglycemic range. As RPG is subject to greater intraindividual variation than other glycemic indicators, we additionally adjusted for fasting time in the model. Though the participants with RPG of 5.6–6.9 mmol/L showed a higher risk of CKD than those with the recommended RPG level (<5.6 mmol/L), our study showed impaired fasting glucose or pre-diabetes was associated with DKD when restricting our analysis to the participants fasting for ≥8 hours.

As far as we know, the present study is the first to explore the association between DM/RPG and the risk of CKD subtypes. The strengths of our study included its prospective study design, large sample size, and long follow-up time. Moreover, the data collection was conducted by well-trained technicians with standardized protocols. Second, we used detailed diabetes information to analyze the impact of DM on the risk of CKD from multiple perspectives, including the source of diabetes diagnosis, diabetes treatment status, and continuum of diabetes. Third, we conducted a series of sensitivity analyses, which helped to avoid the reverse causality and interaction between CKD subtypes.

Inevitably, several limitations should be considered when interpreting the study results. First, we only used diabetes treatment information collected in the baseline survey. However, it may change during follow-up, which was not considered in this study. Second, the CKB cohort collects outcomes from multiple sources, but the majority was from inpatient health insurance. Thus, patients with undiagnosed CKD would be misclassified as participants without diseases of interest in the present study, which leads to an underestimation of the association. Furthermore, it should be acknowledged that the potential for misclassification of CKD subtypes cannot be disregarded when clinical diagnosis primarily relies on manifestations and renal biopsy is not required in routine practice. Third, although we adjusted various established and potential risk factors for CKD, biomarkers (eg, triglycerides, cholesterol) were not measured in the present study, resulting in the possibility of residual confounding. Fourth, RPG level could fluctuate with diet and physical activity during the day. Metrics like HbA1c and glucose trends would better represent how well DM is controlled and managed. Since HbA1c and glucose trends were unavailable in most of the participants in CKB, the external validity of the results may be limited. Fifth, the information on diabetes diagnosis and treatment was self-reported, which might bias the association.

## Conclusion

In summary, we have found positive associations of diabetes with CKD and its specific subtypes, including diabetic nephropathy and glomerular disease in Chinese adults. Patients with diabetes who were undiagnosed or uncontrolled were also associated with an elevated risk of CKD. Even among those who did not have diabetes, elevated blood glucose levels could increase the risk of CKD. Our findings provided evidence that managing diabetes, including early detection, blood glucose monitoring, and control, could reduce the risk of developing CKD.

## Data Availability

Data are available upon reasonable request. The access policy and procedures are available at www.ckbiobank.org.

## References

[1] Karim MA, Kartsonaki C, Bennett DA, et al. Systemic inflammation is associated with incident stroke and heart disease in East Asians. Sci Rep 2020;10:8084. 10.1038/s41598-020-64764-0 32398734 PMC7217894

[2] Ke C, Liang J, Liu M, et al. Burden of chronic kidney disease and its risk-attributable burden in 137 low-and middle-income countries, 1990-2019: results from the global burden of disease study 2019. BMC Nephrol 2022;23:66. 10.1186/s12882-022-02686-x 35164687 PMC8845360

[3] Luyckx VA, Cherney DZI, Bello AK. Preventing CKD in developed countries. Kidney Int Rep 2020;5:263–77. 10.1016/j.ekir.2019.12.003 32154448 PMC7056854

[4] Zhang L, Zhao M-H, Zuo L, et al. China kidney disease network (CK-NET) 2016 annual data report. Kidney Int Suppl 2020;10:e97–185. 10.1016/j.kisu.2020.09.001 PMC7716083 33304640

[5] Kitada M, Koya D. Proposal of classification of 'chronic kidney disease (CKD) with diabetes' in clinical setting. Diabetol Int 2019;10:180–2. 10.1007/s13340-019-00396-8 31275783 PMC6592982

[6] Echouffo-Tcheugui JB, Narayan KM, Weisman D, et al. Association between prediabetes and risk of chronic kidney disease: a systematic review and meta-analysis. Diabet Med 2016;33:1615–24. 10.1111/dme.13113 26997583

[7] Palladino R, Tabak AG, Khunti K, et al. Association between pre-diabetes and microvascular and macrovascular disease in newly diagnosed type 2 diabetes. BMJ Open Diab Res Care 2020;8:e001061. 10.1136/bmjdrc-2019-001061 PMC7202749 32332069

[8] Li Y, Teng D, Shi X, et al. Prevalence of diabetes recorded in Mainland China using 2018 diagnostic criteria from the American Diabetes Association: national cross sectional study. BMJ 2020;369:m997. 10.1136/bmj.m997 32345662 PMC7186854

[9] Chen Z, Chen J, Collins R, et al. China Kadoorie Biobank of 0.5 million people: survey methods, baseline characteristics and long-term follow-up. Int J Epidemiol 2011;40:1652–66. 10.1093/ije/dyr120 22158673 PMC3235021

[10] Chen Z, Lee L, Chen J, et al. Cohort profile: the Kadoorie study of chronic disease in China (KSCDC). Int J Epidemiol 2005;34:1243–9. 10.1093/ije/dyi174 16131516

[11] American Diabetes Association. Classification and diagnosis of diabetes: standards of medical care in diabetes-2021. Diabetes Care 2021;44:S15–33. 10.2337/dc21-S002 33298413

[12] Pang Y, Kartsonaki C, Guo Y, et al. Diabetes, plasma glucose and incidence of colorectal cancer in Chinese adults: a prospective study of 0.5 million people. J Epidemiol Community Health 2018;72:919–25. 10.1136/jech-2018-210651 29970599 PMC6161653

[13] Tohidi M, Hasheminia M, Mohebi R, et al. Incidence of chronic kidney disease and its risk factors, results of over 10 year follow up in an Iranian cohort. PLoS One 2012;7:e45304. 10.1371/journal.pone.0045304 23028919 PMC3459968

[14] Yamagata K, Ishida K, Sairenchi T, et al. Risk factors for chronic kidney disease in a community-based population: a 10-year follow-up study. Kidney Int 2007;71:159–66. 10.1038/sj.ki.5002017 17136030

[15] Fontana F, Perrone R, Giaroni F, et al. Clinical predictors of nondiabetic kidney disease in patients with diabetes: a single-center study. Int J Nephrol 2021;2021:9999621. 10.1155/2021/9999621 34336286 PMC8292077

[16] Zeng Y-Q, Yang Y-X, Guan C-J, et al. Clinical predictors for nondiabetic kidney diseases in patients with type 2 diabetes mellitus: a retrospective study from 2017 to 2021. BMC Endocr Disord 2022;22:168. 10.1186/s12902-022-01082-8 35773653 PMC9248150

[17] Chen S, Chen L, Jiang H. Prognosis and risk factors of chronic kidney disease progression in patients with diabetic kidney disease and non-diabetic kidney disease: a prospective cohort CKD-ROUTE study. Ren Fail 2022;44:1309–18. 10.1080/0886022X.2022.2106872 35938702 PMC9361770

[18] Anders H-J, Huber TB, Isermann B, et al. CKD in diabetes: diabetic kidney disease versus nondiabetic kidney disease. Nat Rev Nephrol 2018;14:361–77. 10.1038/s41581-018-0001-y 29654297

[19] Cho NH, Shaw JE, Karuranga S, et al. IDF diabetes atlas: global estimates of diabetes prevalence for 2017 and projections for 2045. Diabetes Res Clin Pract 2018;138:271–81. 10.1016/j.diabres.2018.02.023 29496507

[20] Gedebjerg A, Almdal TP, Berencsi K, et al. Prevalence of micro- and macrovascular diabetes complications at time of type 2 diabetes diagnosis and associated clinical characteristics: a cross-sectional baseline study of 6958 patients in the Danish DD2 cohort. J Diabetes Complications 2018;32:34–40. 10.1016/j.jdiacomp.2017.09.010 29107454

[21] Tanabe H, Saito H, Machii N, et al. Burden of undiagnosed type 2 diabetes in diabetic kidney disease: a Japanese retrospective cohort study. J Clin Med 2020;9:2028. 10.3390/jcm9072028 32605211 PMC7409199

[22] Wang L, Gao P, Zhang M, et al. Prevalence and ethnic pattern of diabetes and prediabetes in China in 2013. JAMA 2017;317:2515–23. 10.1001/jama.2017.7596 28655017 PMC5815077

[23] Zhang Y, Pan X-F, Chen J, et al. Combined lifestyle factors and risk of incident type 2 diabetes and prognosis among individuals with type 2 diabetes: a systematic review and meta-analysis of prospective cohort studies. Diabetologia 2020;63:21–33. 10.1007/s00125-019-04985-9 31482198

[24] Lin L-K, Sun Y, Heng BH, et al. Medication adherence and glycemic control among newly diagnosed diabetes patients. BMJ Open Diab Res Care 2017;5:e000429. 10.1136/bmjdrc-2017-000429 PMC5574459 28878942

[25] Sun Y, Wang C, Yang W, et al. Fasting blood glucose, but not 2-H postload blood glucose or HbA1C, is associated with mild decline in estimated glomerular filtration rate in healthy Chinese. Int Urol Nephrol 2015;47:147–52. 10.1007/s11255-014-0880-1 25503445

[26] Markus MRP, Ittermann T, Baumeister SE, et al. Prediabetes is associated with microalbuminuria, reduced kidney function and chronic kidney disease in the general population: the KORA (cooperative health research in the Augsburg region) F4-study. Nutr Metab Cardiovasc Dis 2018;28:234–42. 10.1016/j.numecd.2017.12.005 29337019

[27] Bigotte Vieira M, Neves JS, Leitão L, et al. Impaired fasting glucose and chronic kidney disease, albuminuria, or worsening kidney function: a secondary analysis of the SPRINT. J Clin Endocrinol Metab 2019;104:4024–32. 10.1210/jc.2019-00073 31063197 PMC6676073

[28] Kim GS, Oh HH, Kim SH, et al. Association between prediabetes (defined by HbA1(C), fasting plasma glucose, and impaired glucose tolerance) and the development of chronic kidney disease: a 9-year prospective cohort study. BMC Nephrol 2019;20:130. 10.1186/s12882-019-1307-0 30992067 PMC6469043

[29] Koshi T, Sagesaka H, Sato Y, et al. Elevated haemoglobin a1c but not fasting plasma glucose conveys risk of chronic kidney disease in non-diabetic individuals. Diabetes Res Clin Pract 2018;146:233–9. 10.1016/j.diabres.2018.10.026 30391503

