# Supplementary material for: Diabetes and chronic kidney disease in Chinese adults: a population-based cohort study
Source: BMJ Open Diabetes Res Care. 2024 Jan 24;12(1):e003721. doi: 10.1136/bmjdrc-2023-003721 (PMC10823934; doi:10.1136/bmjdrc-2023-003721)
Supplement: Supplementary data [file bmjdrc-2023-003721supp001.pdf]

BMJ Publishing Group Limited (BMJ) disclaims all liability and responsibility arising from any reliance placed on this supplemental material which has been supplied by the author(s)


# Supplementary Materials

Supplementary material for the manuscript entitled “Diabetes and chronic kidney disease in Chinese adults: a population-based cohort study”

Catalogue

Members of the China Kadoorie Biobank collaborative group ........ 2
Supplementary table 1. Classification of CKD subtypes by ICD-10 code. ........ 3
Supplementary table 2. Sensitivity analyses of the association between diabetes and risk of CKD ........ 4
Supplementary table 3. Adjusted HRs for CKD by diabetes status ........ 5
Supplementary table 4. Adjusted HRs for CKD by levels of RPG among participants without diabetes at baseline. ........ 7
Supplementary table 5. Adjusted HRs for CKD by levels of RPG among fasting participants at baseline. ........ 9
Supplementary Figure 1. Flow diagram of categorizing participants into different diabetes status ........ 11
Reference ........ 12

**Members of the China Kadoorie Biobank collaborative group**

**International Steering Committee:** Junshi Chen, Zhengming Chen (PI), Robert Clarke, Rory Collins, Liming Li (PI), Jun Lv, Richard Peto, Robin Walters.

**International Co-ordinating Centre, Oxford:** Daniel Avery, Maxim Barnard, Derrick Bennett, Lazaros Belbasis, Ruth Boxall, Ka Hung Chan, Yiping Chen, Zhengming Chen, Charlotte Clarke, Johnathan Clarke; Robert Clarke, Huaidong Du, Ahmed Edris Mohamed, Hannah Fry, Simon Gilbert, Pek Kei Im, Andri Iona, Maria Kakkoura, Christiana Kartsonaki, Hubert Lam, Kuang Lin, James Liu, Mohsen Mazidi, Iona Millwood, Sam Morris, Qunhua Nie, Alfred Pozarickij, Maryanm Rahmati, Paul Ryder, Saredo Said, Dan Schmidt, Becky Stevens, Iain Turnbull, Robin Walters, Baihan Wang, Lin Wang, Neil Wright, Ling Yang, Xiaoming Yang, Pang Yao.

**National Co-ordinating Centre, Beijing:** Xiao Han, Can Hou, Qingmei Xia, Chao Liu, Jun Lv, Pei Pei, Dianjianyi Sun, Canqing Yu, Lang Pan.

**10 Regional Co-ordinating Centres: Qingdao CDC:** Zengchang Pang, Ruqin Gao, Shanpeng Li, Haiping Duan, Shaojie Wang, Yongmei Liu, Ranran Du, Yajing Zang, Liang Cheng, Xiaocao Tian, Hua Zhang, Yaoming Zhai, Feng Ning, Xiaohui Sun, Feifei Li. **Licang CDC:** Silu Lv, Junzheng Wang, Wei Hou. **Heilongjiang Provincial CDC:** Wei Sun, Shichun Yan, Xiaoming Cui. **Nangang CDC:** Chi Wang, Zhenyuan Wu,Yanjie Li, Quan Kang. **Hainan Provincial CDC:** Huiming Luo, Tingting Ou. **Meilan CDC:** Xiangyang Zheng, Zhendong Guo, Shukuan Wu, Yilei Li, Huimei Li. **Jiangsu Provincial CDC:** Ming Wu, Yonglin Zhou, Jinyi Zhou, Ran Tao, Jie Yang, Jian Su. **Suzhou CDC:** Fang Liu, Jun Zhang, Yihe Hu, Yan Lu, Liangcai Ma, Aiyu Tang, Shuo Zhang, Jianrong Jin, Jingchao Liu. **Guangxi Provincial CDC:** Mei Lin, Zhenzhen Lu. **Liuzhou CDC:** Lifang Zhou, Changping Xie, Jian Lan,Tingping Zhu,Yun Liu, Liuping Wei, Liyuan Zhou, Ningyu Chen, Yulu Qin, Sisi Wang. **Sichuan Provincial CDC:** Xianping Wu, Ningmei Zhang, Xiaofang Chen, Xiaoyu Chang. **Pengzhou CDC:** Mingqiang Yuan, Xia Wu, Xiaofang Chen, Wei Jiang, Jiaqiu Liu, Qiang Sun. **Gansu Provincial CDC:** Faqing Chen, Xiaolan Ren, Caixia Dong. **Maiji CDC:** Hui Zhang, Enke Mao, Xiaoping Wang, Tao Wang, Xi zhang. **Henan Provincial CDC:** Kai Kang, Shixian Feng, Huizi Tian, Lei Fan. **Huixian CDC:** XiaoLin Li, Huarong Sun, Pan He, Xukui Zhang. **Zhejiang Provincial CDC:** Min Yu, Ruying Hu, Hao Wang. **Tongxiang CDC**: Xiaoyi Zhang, Yuan Cao, Kaixu Xie, Lingli Chen, Dun Shen. **Hunan Provincial CDC:** Xiaojun Li, Donghui Jin, Li Yin, Huilin Liu, Zhongxi Fu. **Liuyang CDC:** Xin Xu, Hao Zhang, Jianwei Chen,Yuan Peng, Libo Zhang, Chan Qu.

**Supplementary table 1. Classification of CKD subtypes by ICD-10 code.**

| CKD subtypes | ICD-10 | | | | | |
|---|---|---|---|---|---|---|
| Diabetic nephropathy | E10.2 | E11.2 | E12.200 | E13.2 | E14.2 | N08.3 |
| Glomerular disease | N02 | N03 | N04 | N05 | N06 | N39.1 |
| Hypertensive renal disease | I12 | I13 | O10.200 | O10.201 | O10.300 | O10.301 |
| | O11.00 | O11.01 | | | | |
| Renal tubulointerstitial diseases | N11 | N12 | N14 | N15.000 | N15.001 | N15.800 |
| | N15.801 | N15.900 | N16 | N25.1 | N25.8 | N25.9 |
| | N28.905 | M10.001 | M32.102 | M32.113 | M35.006 | M35.005 |
| | I15.101 | E72.0 | E74.8 | | | |
| Obstructive nephropathy | N13.0 | N13.1 | N13.2 | N13.8 | | |
| Other subtypes | N07 | N08 | N28.0 | N28.8 | N28.9 | N25.0 |
| | N05.9 | N16.4 | N29.8 | N22.8 | N26 | M10.005 |
| | M10.300 | M10.391 | M10.392 | M10.393 | M31.001 | M31.002 |
| | M31.003 | M31.005 | M31.102 | M31.303 | M31.4 | M31.700 |
| | M31.701 | M31.802 | M32.101 | M32.105 | M32.102 | M32.112 |
| | M32.113 | Q60 | Q61.1 | Q61.2 | Q61.3 | Q61.5 |
| | Q63.1 | Q63.9 | A52.712 | P96.0 | R39.2 | I15.0 |
| | I77.604 | T86.1 | D59.3 | D69.005 | Z49 | O26.801 |
| | O26.811 | O26.804 | O26.812 | O26.802 | O26.813 | B18.103 |
| | B18.102 | B18.205 | B18.208 | D89.101 | E85.002 | E85.003 |
| | E85.411 | E85.410 | L40.803 | L40.802 | | |
| | E10.2 | E11.2 | E12.200 | E13.2 | E14.2 | N08.3 |
| | I12 | I13 | O10.200 | O10.201 | O10.300 | O10.301 |

CKD: chronic kidney disease; ICD-10: the International Classification of Diseases, Tenth Revision.

**Supplementary table 2. Sensitivity analyses of the association between diabetes and risk of CKD**

| | Additionally adjusting diabetes treatment | Excluding the early two years of follow-up | Censored at first-onset of CKD subtypes |
|---|---|---|---|
| CKD | | | |
| No diabetes | 1.00 | 1.00 | - |
| Diabetes | 3.06 (2.78-3.37) | 4.57 (4.27-4.89) | - |
| Diabetic nephropathy | | | |
| No diabetes | 1.00 | 1.00 | 1.00 |
| Diabetes | 20.04 (16.98-23.66) | 33.31 (29.03-38.24) | 33.34 (28.89-38.47) |
| Glomerulonephritis | | | |
| No diabetes | 1.00 | 1.00 | 1.00 |
| Diabetes | 1.65 (1.33-2.06) | 1.65 (1.38-1.97) | 1.51 (1.25-1.84) |
| Hypertensive nephropathy | | | |
| No diabetes | 1.00 | 1.00 | 1.00 |
| Diabetes | 1.24 (0.80-1.93) | 1.40 (1.01-1.95) | 1.20 (0.82-1.75) |
| Tubulointerstitial nephritis | | | |
| No diabetes | 1.00 | 1.00 | 1.00 |
| Diabetes | 1.11 (0.71-1.75) | 1.34 (0.95-1.88) | 1.36 (0.97-1.90) |
| Obstructive nephropathy | | | |
| No diabetes | 1.00 | 1.00 | 1.00 |
| Diabetes | 0.79 (0.46-1.38) | 0.86 (0.55-1.34) | 0.86 (0.55-1.34) |

HRs were stratified by baseline age groups and study regions and adjusted for education, household income, occupation, alcohol consumption, smoking, met, BMI, and hypertension status.

CKD: chronic kidney disease; HR: hazard ratio; BMI, body mass index.

**Supplementary table 3. Adjusted HRs for CKD by diabetes status**

| | No. of events | Rates[a] | HR (95%CI) |
|---|---|---|---|
| **CKD** | | | |
| No diabetes | 4,116 | 78.28 | 1.00 |
| Screened diabetes | 396 | 279.53 | 2.83 (2.55-3.15) |
| Previously diagnosed diabetes | | | |
| No medication | | | |
| Controlled[b] | 26 | 222.24 | 2.21 (1.50-3.25) |
| Uncontrolled[c] | 74 | 739.94 | 7.09 (5.63-8.94) |
| Under medication | | | |
| Controlled[b] | 216 | 471.29 | 4.27 (3.71-4.91) |
| Uncontrolled[c] | 589 | 861.74 | 8.17 (7.46-8.95) |
| **Diabetic nephropathy** | | | |
| No diabetes | 348 | 6.6 | 1.00 |
| Screened diabetes | 204 | 143.37 | 17.35 (14.50-20.77) |
| Previously diagnosed diabetes | | | |
| No medication | | | |
| Controlled[b] | 15 | 127.85 | 15.88 (9.44-26.72) |
| Uncontrolled[c] | 54 | 536.53 | 60.41 (45.03-81.04) |
| Under medication | | | |
| Controlled[b] | 147 | 319.27 | 37.37 (30.48-45.81) |
| Uncontrolled[c] | 404 | 586.46 | 68.30 (58.40-79.88) |
| **Glomerulonephritis** | | | |
| No diabetes | 1,372 | 26.05 | 1.00 |
| Screened diabetes | 73 | 51.23 | 1.60 (1.26-2.03) |
| Previously diagnosed diabetes | | | |
| No medication | | | |
| Controlled[b] | 5 | 42.56 | 1.24 (0.52-2.99) |
| Uncontrolled[c] | 10 | 98.78 | 2.92 (1.56-5.44) |
| Under medication | | | |
| Controlled[b] | 18 | 38.79 | 1.03 (0.65-1.65) |
| Uncontrolled[c] | 53 | 75.93 | 2.13 (1.61-2.82) |
| **Hypertensive nephropathy** | | | |
| No diabetes | 320 | 6.07 | 1.00 |
| Screened diabetes | 20 | 14.01 | 1.31 (0.83-2.06) |
| Previously diagnosed diabetes | | | |
| No medication | | | |
| Controlled[b] | 1 | 8.51 | 0.79 (0.11-5.63) |
| Uncontrolled[c] | 1 | 9.85 | 0.92 (0.13-6.54) |
| Under medication | | | |

| | No. of events | Rates[a] | HR (95%CI) |
|---|---|---|---|
| Controlled[b] | 7 | 15.07 | 1.23 (0.58-2.61) |
| Uncontrolled[c] | 15 | 21.46 | 1.73 (1.02-2.94) |
| **Tubulointerstitial nephritis** | | | |
| No diabetes | 437 | 8.29 | 1.00 |
| Screened diabetes | 18 | 12.62 | 1.15 (0.72-1.86) |
| Previously diagnosed diabetes | | | |
| No medication | | | |
| Controlled[b] | 2 | 17.02 | 1.62 (0.40-6.53) |
| Uncontrolled[c] | 0 | 0 | - |
| Under medication | | | |
| Controlled[b] | 10 | 21.55 | 1.80 (0.95-3.38) |
| Uncontrolled[c] | 13 | 18.61 | 1.53 (0.88-2.68) |
| **Obstructive nephropathy** | | | |
| No diabetes | 573 | 10.87 | 1.00 |
| Screened diabetes | 11 | 7.71 | 0.76 (0.42-1.38) |
| Previously diagnosed diabetes | | | |
| No medication | | | |
| Controlled[b] | 0 | 0 | - |
| Uncontrolled[c] | 2 | 19.73 | 2.16 (0.54-8.70) |
| Under medication | | | |
| Controlled[b] | 2 | 4.31 | 0.54 (0.13-2.16) |
| Uncontrolled[c] | 7 | 10.02 | 1.26 (0.60-2.68) |

HRs were stratified by study regions and age at the study date and adjusted for sex, education, household income, occupation, alcohol consumption, smoking consumption, met, BMI, and hypertension status.

[a] The incidence rate was derived from CKD case count divided by total person-years (presented as per 100,000 person-years). Total person-year at risk was calculated from the time participants completed the baseline survey until the occurrence of CKD, death, loss to follow-up, or Dec 31st, 2018, whichever came first.

[b] Those without screen-detected diabetes were defined as the controlled group.

[c] Those who met screen-detected diabetes criteria were defined as the uncontrolled group.

HR: hazard ratio; CKD: chronic kidney disease; BMI, body mass index.

**Supplementary table 4. Adjusted HRs for CKD by levels of RPG among participants without diabetes at baseline.**

| | No. of events | Rates[a] | HR (95%CI) |
|---|---|---|---|
| CKD (mmol/L) | | | |
| <5.6 | 2,021 | 67.37 | 1.00 |
| 5.6-6.9 | 1,411 | 85.04 | 1.12 (1.05-1.20) |
| 7.0-11.0 | 684 | 114.21 | 1.38 (1.26-1.51) |
| p for trend | | | <0.001 |
| Effects per 1 mmol/L | | | 1.11 (1.08-1.14) |
| Diabetic nephropathy | | | |
| <5.6 | 122 | 4.06 | 1.00 |
| 5.6-6.9 | 123 | 7.39 | 1.51 (1.17-1.95) |
| 7.0-11.0 | 103 | 17.15 | 2.92 (2.22-3.83) |
| p for trend | | | <0.001 |
| Effects per 1 mmol/L | | | 1.40 (1.30-1.50) |
| Glomerulonephritis | | | |
| <5.6 | 709 | 23.60 | 1.00 |
| 5.6-6.9 | 443 | 26.65 | 1.10 (0.97-1.24) |
| 7.0-11.0 | 220 | 36.64 | 1.45 (1.24-1.70) |
| p for trend | | | <0.001 |
| Effects per 1 mmol/L | | | 1.12 (1.07-1.17) |
| Hypertensive nephropathy | | | |
| <5.6 | 148 | 4.92 | 1.00 |
| 5.6-6.9 | 109 | 6.55 | 1.07 (0.83-1.38) |
| 7.0-11.0 | 63 | 10.49 | 1.37 (1.01-1.86) |
| p for trend | | | 0.049 |
| Effects per 1 mmol/L | | | 1.09 (1.00-1.19) |
| Tubulointerstitial nephritis | | | |
| <5.6 | 238 | 7.92 | 1.00 |
| 5.6-6.9 | 148 | 8.90 | 0.94 (0.76-1.16) |
| 7.0-11.0 | 51 | 8.49 | 0.92 (0.67-1.25) |
| p for trend | | | 0.514 |
| Effects per 1 mmol/L | | | 0.94 (0.86-1.03) |
| Obstructive nephropathy | | | |
| <5.6 | 295 | 9.81 | 1.00 |
| 5.6-6.9 | 203 | 12.20 | 1.14 (0.95-1.36) |
| 7.0-11.0 | 75 | 12.48 | 1.12 (0.86-1.45) |
| p for trend | | | 0.242 |
| Effects per 1 mmol/L | | | 1.05 (0.98-1.13) |

HRs were stratified by baseline age groups and study regions and adjusted for education, household income, occupation, fasting time, alcohol consumption, smoking consumption, met, BMI, and hypertension status.

[a] The incidence rate was derived from CKD case count divided by total person-years (presented as per 100,000 person-years). Total person-year at risk was calculated from the time participants completed the baseline survey until the occurrence of CKD, death, loss to

follow-up, or Dec 31st, 2018, whichever came first.

HR: hazard ratio; CKD: chronic kidney disease; RPG, random plasma glucose; BMI, body mass index.

**Supplementary table 5. Adjusted HRs for CKD by levels of RPG among fasting participants at baseline.**

| | No. of events | Incidence Rate[a] | HR (95% CI) |
|---|---|---|---|
| CKD (mmol/L) | | | |
| <5.6 | 616 | 74.42 | 1.00 |
| 5.6-6.9[b] | 280 | 95.96 | 1.14 (0.99-1.32) |
| 7.0-11.0 | 154 | 257.88 | 2.45 (2.04-2.95) |
| ≥11.1 | 138 | 837.57 | 8.61 (7.10-10.45) |
| p for trend | | | <0.001 |
| Effect per 1 mmol/L | | | 1.22 (1.20-1.24) |
| Diabetic nephropathy | | | |
| <5.6 | 69 | 8.31 | 1.00 |
| 5.6-6.9[b] | 53 | 18.11 | 1.93 (1.34-2.78) |
| 7.0-11.0 | 90 | 149.97 | 11.63 (8.37-16.16) |
| ≥11.1 | 91 | 548.25 | 46.15 (33.08-64.40) |
| p for trend | | | <0.001 |
| Effects per 1 mmol/L | | | 1.34 (1.31-1.37) |
| Glomerulonephritis | | | |
| <5.6 | 248 | 29.92 | 1.00 |
| 5.6-6.9[b] | 96 | 32.82 | 1.02 (0.80-1.29) |
| 7.0-11.0 | 26 | 43.18 | 1.13 (0.75-1.71) |
| ≥11.1 | 17 | 101.21 | 2.98 (1.80-4.92) |
| p for trend | | | <0.001 |
| Effects per 1 mmol/L | | | 1.12 (1.08-1.17) |
| Hypertensive nephropathy | | | |
| <5.6 | 35 | 4.22 | 1.00 |
| 5.6-6.9[b] | 24 | 8.20 | 1.33 (0.78-2.28) |
| 7.0-11.0 | 8 | 13.26 | 1.46 (0.66-3.23) |
| ≥11.1 | 2 | 11.88 | 1.28 (0.30-5.44) |
| p for trend | | | 0.500 |
| Effects per 1 mmol/L | | | 1.08 (0.96-1.20) |
| Tubulointerstitial nephritis | | | |
| <5.6 | 74 | 8.92 | 1.00 |
| 5.6-6.9[b] | 31 | 10.59 | 1.02 (0.67-1.57) |
| 7.0-11.0 | 7 | 11.61 | 1.03 (0.47-2.27) |
| ≥11.1 | 5 | 29.74 | 2.90 (1.15-7.28) |
| p for trend | | | 0.046 |
| Effects per 1 mmol/L | | | 1.08 (0.98-1.19) |
| Obstructive nephropathy | | | |
| <5.6 | 51 | 6.14 | Ref. |
| 5.6-6.9[b] | 19 | 6.49 | 1.16 (0.68-1.99) |
| 7.0-11.0 | 6 | 9.95 | 1.64 (0.69-3.94) |
| ≥11.1 | 1 | 5.94 | 1.14 (0.16-8.37) |

| | No. of events | Incidence Rate[a] | HR (95% CI) |
|---|---|---|---|
| p for trend | | | 0.477 |
| Effects per 1 mmol/L | | | 1.07 (0.93-1.22) |

HRs were stratified by baseline age groups and study regions and adjusted for education, household income, occupation, fasting time, alcohol consumption, smoking consumption, met, BMI, and hypertension status.

[a] The incidence rate was derived from CKD case count divided by total person-years (presented as per 100,000 person-years). Total person-year at risk was calculated from the time participants completed the baseline survey until the occurrence of CKD, death, loss to follow-up, or Dec 31st, 2018, whichever came first.

[b] The group (RPG 5.6-6.9 mmol/L) was defined as prediabetes according to the criteria proposed by the American Diabetes Association.[1]

HR: hazard ratio; CKD: chronic kidney disease; RPG, random plasma glucose; BMI, body mass index.

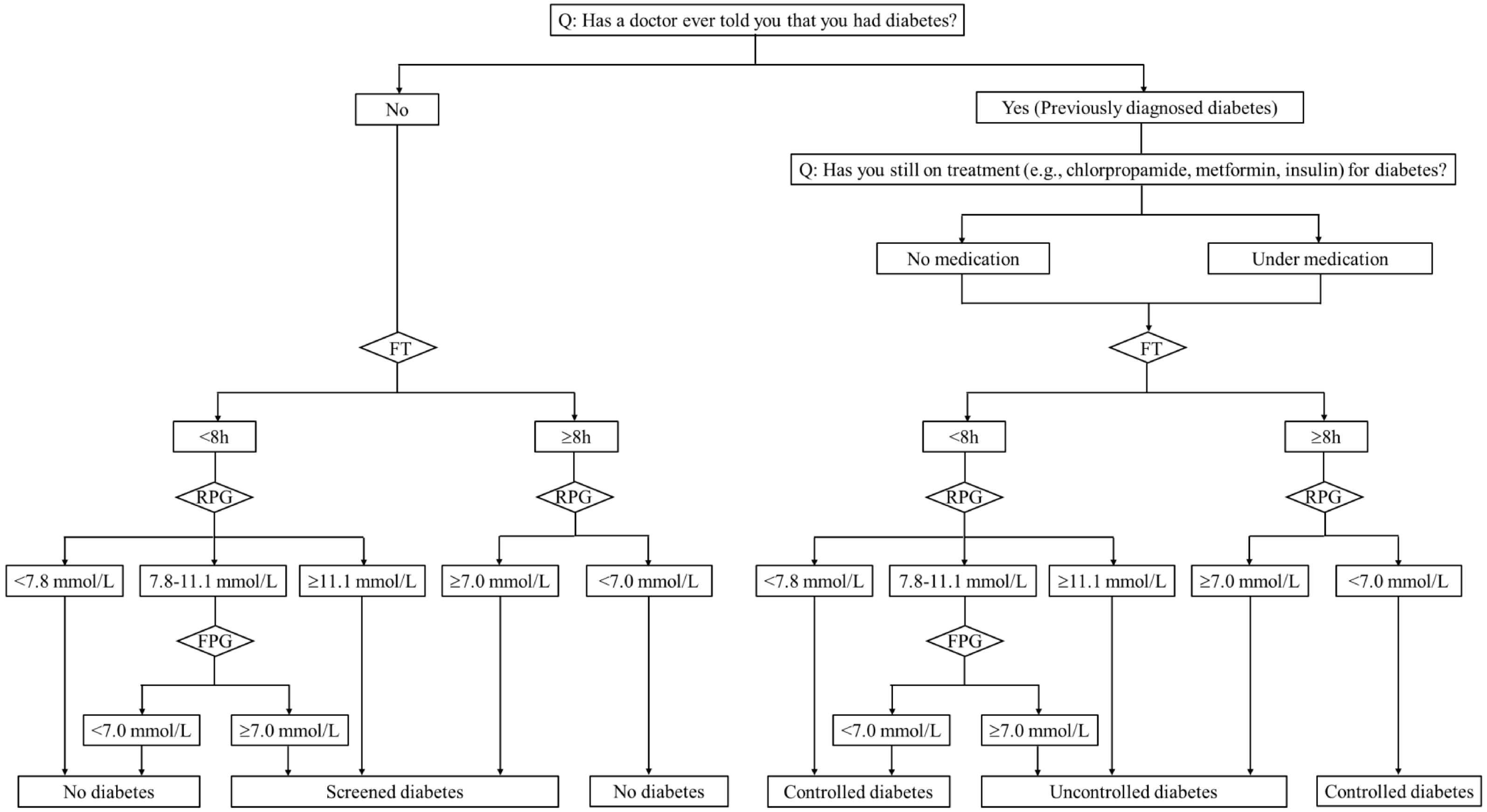


**Supplementary Figure 1. Flow diagram of categorizing participants into different diabetes status**

FT, fasting time; RPG, random plasma glucose; FPG, fasting plasma glucose

**Reference**

1. American Diabetes Association. 2. Classification and Diagnosis of Diabetes: Standards of Medical Care in Diabetes-2021. *Diabetes Care* 2021(44(Suppl 1)):S15-S33. doi: 10.2337/dc21-S002
